# Metabolizable energy requirement for maintenance estimated by regression analysis of body weight gain or metabolizable energy intake in growing pigs

**DOI:** 10.5713/ajas.17.0898

**Published:** 2019-02-07

**Authors:** Hu Liu, Yifan Chen, Zhongchao Li, Yakui Li, Changhua Lai, Xiangshu Piao, Jaap van Milgen, Fenglai Wang

**Affiliations:** 1 State Key Laboratory of Animal Nutrition, College of Animal Science and Technology, China Agricultural University, Beijing 100193, China; 2 INRA, UMR Pegase, 35590 Saint-Gilles, France

**Keywords:** Fat, Growing Pigs, Indirect Calorimetry, Maintenance Energy Requirement, Protein

## Abstract

**Objective:**

Feed energy required for pigs is first prioritized to meet maintenance costs. Additional energy intake in excess of the energy requirement for maintenance is retained as protein and fat in the body, leading to weight gain. The objective of this study was to estimate the metabolizable energy requirements for maintenance (ME_m_) by regressing body weight (BW) gain against metabolizable energy intake (MEI) in growing pigs.

**Methods:**

Thirty-six growing pigs (26.3±1.7 kg) were allotted to 1 of 6 treatments with 6 replicates per treatment in a randomized complete block design. Treatments were 6 feeding levels which were calculated as 50%, 60%, 70%, 80%, 90%, or 100% of the estimated *ad libitum* MEI (2,400 kJ/kg BW^0.60^ d). All pigs were individually housed in metabolism crates for 30 d and weighed every 5 d. Moreover, each pig from each treatment was placed in the open-circuit respiration chambers to measure heat production (HP) and energy retained as protein (RE_p_) and fat (RE_f_) every 5 d. Serum biochemical parameters of pigs were analyzed at the end of the experiment.

**Results:**

The average daily gain (ADG) and HP as well as the RE_p_ and RE_f_ linearly increased with increasing feed intake (p<0.010). β-hydroxybutyrate concentration of serum tended to increase with increasing feed intake (p = 0.080). The regression equations of MEI on ADG were MEI, kJ/kg BW^0.60^ d = 1.88×ADG, g/d+782 (R^2^ = 0.86) and ME_m_ was estimated at 782 kJ/kg BW^0.60^ d. Protein retention of growing pigs would be positive while RE_f_ would be negative at this feeding level via regression equations of RE_p_ and RE_f_ on MEI.

**Conclusion:**

The ME_m_ was estimated at 782 kJ/kg BW^0.60^ d in current experiment. Furthermore, growing pigs will deposit protein and oxidize fat if provided feed at the estimated maintenance level.

## INTRODUCTION

Feed energy required for pigs is first prioritized to meet maintenance costs [1]. Maintenance comprises the basal energy requirements for supporting body function, body temperature and necessary activity at a time when there is no net gain or loss of tissue [2]. Maintenance energy requirement should be independent of the animal’s production state and nutritional levels, and therefore, it should be related to animal characteristics only [3]. Furthermore, the energy requirement for maintenance is an important part of net energy (NE) system, and accuracy of estimation of the energy requirements for maintenance will influence the absolute NE value of a feed ingredient [4–6].

Energy requirements for maintenance can be estimated us ing body weight (BW) gain as an indirect index of energy retention by the regression analysis method [4,5]. Yuliarty et al [6] reported that metabolizable energy requirements for maintenance (ME_m_) of entire male Bali cattle in East Timor determined by regressing BW change against metabolizable energy intake (MEI). Compared to the method of determination the heat leaving the animal’s body, one advantage of this method is that the measurement of BW can be very precise [7]. Moreover, compared with fasting method, measurements can be obtained in a relatively healthy physiological state, which might decrease variability in the energy concentration of tissue gain [7].

Numerous studies determined the effects of various MEI levels on heat production (HP) by graded feed intake of identical diet [3,8]. However, increasing feed intake increased not only MEI, but also other nutrients as protein and fat. Thus, the effects of feeding level on digestibility and nitrogen and energy balances in growing pigs were determined [9,10]. Furthermore, additional energy intake in excess of the ME_m_ is retained as protein (RE_p_) and fat (RE_f_) in the body [11]. However, little is known of the effects of feeding level on the energy retention for protein and fat in growing pigs. A series of studies have demonstrated that feeding level plays a role in blood biochemistry [12]. The serum biochemical parameters related to lipid metabolism and protein metabolism could be reflected the energy deposition and mobilization [13].

Therefore, the objective of this experiment was to estimate ME_m_ in growing pigs by regressing BW gain against MEI. In addition, the effects of feeding level on the nitrogen and energy balance were also measured.

## MATERIALS AND METHODS

The experimental protocol used in the present study was approved by the Institutional Animal Care and Use Committee at China Agricultural University (Beijing, China).

### Equipment

To determine the components of energy metabolism, 6 open-circuit respiration chambers (7.8 m^3^ in volume) were used. The design of the chambers was previously reported by Zhang et al [14]. By means of a gas-tight ventilator, fresh air was drawn into the chambers where it was thoroughly mixed with the air in the chamber. The chambers were air-conditioned to maintain a constant temperature of 22°C at approximately 70% relative humidity. Temperature and atmospheric pressure in the chamber were measured and used to calculate gas extraction rate under standard temperature (0°C) and pressure (101 kPa). Concentrations of oxygen inside and outside the chamber were measured with a Paramagnetic Differential Gas Analyzer (Oxymat 6E, Siemens, Munich, Germany), and concentrations of CO_2_, CH_4_, and NH_3_ were measured with Infrared Gas Analyzers (Ultramat 6E, Siemens, Germany).

All measurements of airflow, gas composition, and climatic conditions in the chambers were conducted at 5-min intervals for calculations of gas exchange. Two respiration chambers shared one gas analyzer. Analyzers had a range of determination of 19.5% to 21% for O_2_, 0% to 1% for CO_2_, 0% to 0.1% for CH_4_, and 0% to 0.1% for NH_3_ with a sensitivity of 0.2% within the determination range. The airflow of extraction was measured by a Mass Flow Meter (Alicat, Tucson, AZ, USA).

### Animals, diets and experimental design

Thirty-six growing barrows (Duroc×Landrace×Yorkshire) with an average initial BW of 26.3±1.7 kg were selected from the Fengning Swine Research farm of China Agricultural University (Hebei, China), and the experiment was conducted for 30 d. Pigs were stratified by BW into 6 blocks of 6 pigs for each treatment. Block 1 comprised the heaviest 6 pigs while block 6 comprised the lightest 6 pigs. Within each block, the pigs were randomly assigned to 1 of 6 feeding levels in a randomized complete block design to give 6 replicates per level for the entire experiment. The feeding levels represented a targeted daily intake of 50%, 60%, 70%, 80%, 90%, or 100% of their estimated *ad libitum* MEI (2,400 kJ/kg BW^0.60^ d) [14,15]. The basal diet was formulated based on corn and soybean meal for growing pigs (Table 1).

The experiment in the respiration chambers was conducted in 6 periods of 5 d. During each period, 6 pigs chosen from 6 feeding level were placed in the open-circuit respiration chambers. Pigs assigned in the same block were placed in the respiration chambers at the same period. Therefore, 6 pigs in block 1 were placed in the chamber during the first period, and 6 pigs in block 6 were placed in the chamber during the last period. This procedure was chosen to minimize the effects of BW on the parameters measured.

Pigs and given feeds were weighed at the start and end of each period to calculate average daily gain (ADG) and to determine the actual amount of feed consumed during each period. Body weight gain and feed consumption were used to estimate gain-to-feed ratio (G:F).

All pigs were individually housed in stainless steel meta bolic crates during the entire 30 d experiment. During the time that pigs were not in the respiration chambers, they were housed in an adjacent room under similar environmental conditions as those in the respiration chambers.

All pigs received their assigned feeding level throughout the 30 d experiment. Pigs were fed an equal amount of meal twice daily at 0900 and 1530 with free access to water. The actual amounts of feed were based on the body weight of pigs at the start of each period. Feed refusals and spillage were recorded daily.

### Sample collection

Feces and urine were collected only during the period when pigs were in the respiration chambers according to the methods described by Liu et al [8]. Feces samples were sealed in plastic bags and stored at −20°C. Urine was collected every morning for each pig into plastic buckets containing 50 mL of 6 *N* HCl and sieved with cotton gauze and filtered into a plastic bottle every day. The total quantity of collected urine was weighed, and 5% of the daily urinary excretion was stored at −20°C.

At the end of the experiment, feces and urine were thawed and separately mixed for each animal and a sub-sample was collected for analysis. Fecal samples were oven-dried for 72 h at 65°C. The feed and dried fecal samples were ground through a 1-mm screen and mixed thoroughly for chemical analysis. A 4 mL urine sample was dripped on to 2 filter papers in a special crucible and dried for 8 h at 65°C in a drying oven.

Concentrations of O _2_, CO_2_, and CH_4_ in ingoing and outgoing air, and outgoing air flow rates were measured during the period when pigs were placed in the respiration chambers. These values were used to calculate O_2_ consumption and CO_2_ and CH_4_ production.

Blood samples were collected from each pig via the ante rior vena cava into 10-mL tubes containing no anticoagulant (Becton Dickinson Vacutainer Systems, Franklin Lakes, NJ, USA) at the end of the experiment following a 12-h fast. Samples were centrifuged (Biofuge22R; Heraeus, Hanau, Germany) at 3,000×*g* for 10 min, and the serum was stored at −80°C until analyzed.

### Chemical analysis

All chemical analyses were conducted in duplicate. Samples of ingredients, diets and feces were analyzed for dry matter (DM, method 930.15, AOAC [16]), crude protein (CP, method 984.13, AOAC [16]), crude fiber (method 978.10, AOAC [16]), calcium (method 927.02, AOAC [16]), total phosphorus (method 984.27, AOAC [16]), and ether extract (Thiex et al [17]). Gross energy in diets, feces and urine were measured using an isoperibol bomb calorimeter (Parr 6400 Calorimeter, Moline, IL, USA) according to Zhang et al [14].

Analysis of amino acid (AA) content in the ingredients and diets was conducted according to Li et al [18]. For most AA, not including methionine, cysteine and tryptophan, samples were analyzed after 6 *N* HCl hydrolysis for 24 h at 110°C using an amino acid analyzer (Hitachi L-8800, Hitachi, Ltd., Tokyo, Japan). Methionine and cysteine were determined as methionine sulfone and cysteic acid, respectively, after cold performic acid oxidation overnight and hydrolysis with 7.5 *N* HCl for 24 h at 110°C. Tryptophan was analyzed after LiOH hydrolysis for 22 h at 110°C using high-performance liquid chromatography (Agilent 1200 Series; Agilent Technologies Incorporated, Santa Clara, CA, USA).

After the frozen serum samples were thawed at 4°C, serum concentrations of glucose, serum urea nitrogen (SUN), creatinine, triglyceride, free fatty acids (FFA) and β-hydroxybutyric acid (BHBA) were quantified using an automatic biochemical analyzer (Hitachi 7160, Hitachi, Ltd., Japan) at the Beijing Sino-UK institute of Biological Technology (Beijing, China). Leptin concentration was assayed using a radioimmunoassay method following the manufacturer’s instructions (Sino-UK institute of Biological Technology, China).

### Calculations

The apparent total tract digestibilities (ATTD) of DM, CP, and gross energy (GE) were calculated according to standard procedures [19]. The digestible energy (DE) content of diet was calculated as the difference between GE intake and the energy lost in feces. Methane loss was negligible and therefore was ignored when the ME content was determined which corresponded to the difference between DE and the energy lost in urine [19].

The HP, non-protein respiratory quotient (RQ_np_), and RQ were calculated daily from O_2_ consumption, as well as CO_2_ and CH_4_ production and nitrogen excretion in urine (UN) during the 5-d balance period according to the following formulas by Brouwer et al [20]:

$$\begin{array}{l} \text{HP }(\text{kJ})=16.1753\times {\text{O}}_{2}\;(\text{L})+5.0208\times {\text{CO}}_{2}\;(\text{L})-2.1673\times {\text{CH}}_{4}\;(\text{L})-5.9873\times \text{UN }(\text{g})\\ {\text{RQ}}_{\text{np}}=[({\text{CO}}_{2}\;(\text{L})-\text{UN }(\text{g})\times 6.25\times 0.774)/({\text{O}}_{2}\;(\text{L})-\text{UN }(\text{g})\times 6.25\times 0.957)]\\ \text{RQ}={\text{CO}}_{2}\;(\text{L})/{\text{O}}_{2}\;(\text{L}) \end{array}$$

Oxidation of protein (OXP) and oxidation of carbohydrate (OXCHO) were calculated by the method described by Chwalibog et al [21] and validated for RQ_np_ values above and below 1.00 by Chwalibog et al [22] as:

$$\begin{array}{l} \text{OXP}\;(\text{kJ})=\text{UN}\;(g)\times 6.25\times 18.42\\ \text{OXCHO }(\text{kJ})=[-2.968\times {\text{O}}_{2}\;(\text{L})+4.147\times {\text{CO}}_{2}\;(\text{L})-1.761\times {\text{CH}}_{4}\;(\text{L})-2.446\times \text{UN }(\text{g})]\times 17.58 \end{array}$$

Energy retention was calculated as the difference between daily MEI and mean HP during the 5-d balance period. Energy retained as protein was calculated as nitrogen retention (g)×6.25×23.8 (kJ/g) according to Chwalibog et al [23]. Energy retained as fat was calculated as the difference between total energy retention and the energy retained as protein.

### Statistical analysis

Data were analyzed using PROC general linear model procedure in SAS (SAS Institute Inc., Cary, NC, USA) as a randomized complete block design. The individual pig was used as the experimental unit for all response variables in the model, which included feeding level as the main effect. Orthogonal polynomial contrasts were used to examine linear and quadratic effects of feeding level on growth performance, ATTD values, energy, and nitrogen balance in growing pigs. Differences were considered significant at p≤0.05, whereas tendencies were discussed at p>0.05 but p≤0.10. Linear regression analyses were conducted by OriginPro 8.5 (OriginLab, Northampton, MA, USA) to determine the relationship between ADG (g), RE_p_ (g/d) or RE_f_ (g/d) and MEI (kJ/kg BW^0.60^ d).

## RESULTS

### Growth performance and nutrient utilization

All pigs were healthy, and the experiment was carried out with all animals in a normal physiological state. The effects of feeding level on growth performance, ATTD of DM, GE, and CP and energy values of the diet are presented in Table 2. The final BW and ADG linearly increased as feeding level increases (p<0.010). The G:F improved linearly with increase in feed intake (p = 0.025). However, there were no difference for ATTD of DM, GE, and CP with increasing dietary energy intake. The DE and ME content had no association with feeding level. There was little evidence of an association between ME:DE ratio and level of dietary energy intake.

### Energy and nitrogen balance

**Figure 1:**
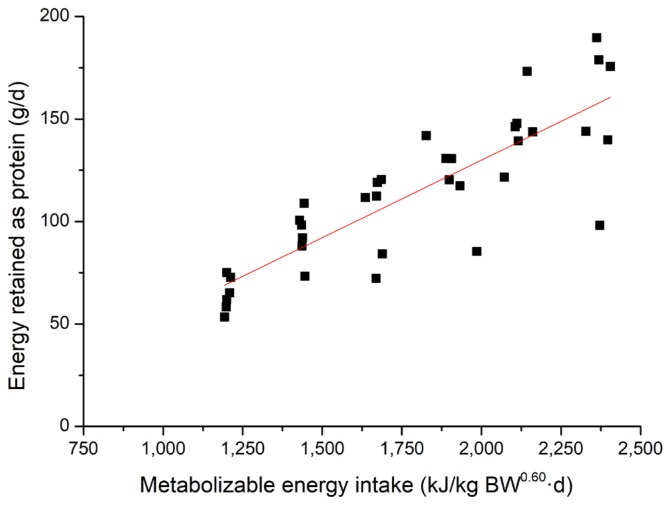
Linear relationship (parameters±standard error) between metabolizable energy intake (MEI) and energy retained as protein (RE_p_) for all pigs (■). RE_p_, g/d = 0.076 (±0.008) ×MEI, kJ/kg BW^0.60^ d–21.28 (±15.286), R^2^ = 0.71, p<0.01, n = 36.

**Figure 2:**
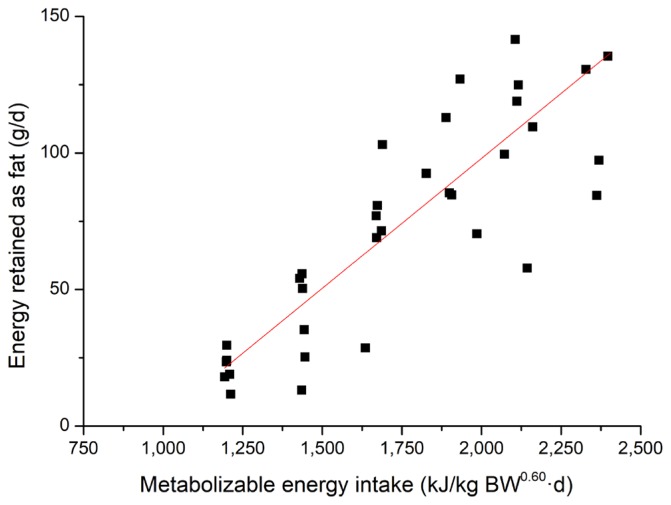
Linear relationship (parameters±standard error) between metabolizable energy intake (MEI) and energy retained as fat (RE_f_) for all pigs (■). RE_f_, g/d = 0.095 (±0.009)×MEI, kJ/kg BW^0.60^ d –92.21 (±17.189), R^2^ = 0.75, p<0.01, n = 36.

The effects of feeding level on energy and nitrogen balance and energy retention in growing pigs are shown in Table 3. There was a linear increase in total HP from 953 kJ/kg BW^0.60^ d to 1,266 kJ/kg BW^0.60^ d as feeding level increases (p = 0.001) from 50% to 100% of *ad libitum* intake. When the total HP was partitioned into heat production from OXP and OXCHO, the OXP had no association with increasing dietary energy intake. However, for OXCHO, a linear increase was observed as feeding level increases (p = 0.023). Nitrogen intake and excreted in feces linearly increased (p<0.010) as dietary ME increases. However, nitrogen excreted in urine had no association with dietary energy. Nitrogen retention also showed a positive linear response (p = 0.002) with increasing levels of feed intake. Energy retention, when expressed as protein, fat or total, linearly increased as feeding level increases (p<0.001). To determine the effect of feeding level on the energy retention for fat and protein in growing pigs, linear regressions of REp and REf in growing pigs were performed and presented in Figure 1 and 2. The equations (parameters±standard error [SE]) for RE_p_ and RE_f_ were RE_p_, g/d = 0.076 (±0.008)×MEI, kJ/kg BW^0.60^ d–21.28 (±15.286) (R^2^ = 0.71, p<0.001) and RE_f_, g/d = 0.095 (±0.009)× MEI, kJ/kg BW^0.60^ d–92.21 (±17.189) (R^2^ = 0.75, p<0.001), respectively. A linear response was observed for the RQ and RQ_np_ as MEI increases (p<0.010).

### Serum biochemical parameters related to energy metabolism

The effects of feeding level on the concentration of serum biochemical parameters in growing pigs are shown in Table 4. The BHBA concentration tended to increase with increasing feed intake (p = 0.080). However, glucose, SUN, creatinine, triglyceride, FFA, and leptin concentrations showed little association with dietary energy intake.

### Energy requirement for maintenance

**Figure 3:**
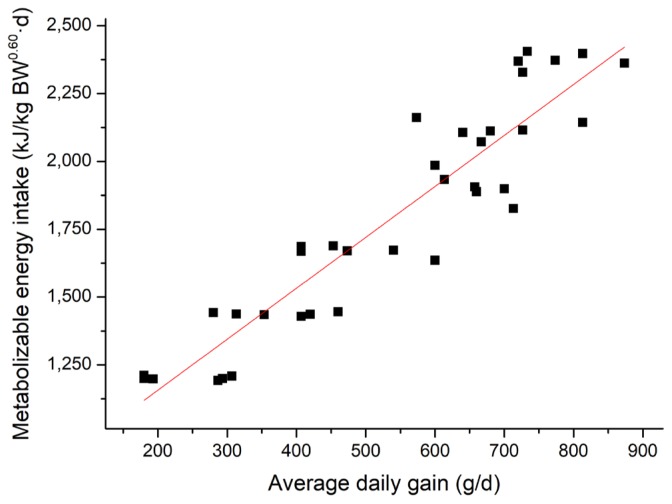
Linear relationship (parameters±standard error) between metabolizable energy intake (MEI) and average daily gain (ADG) for all pigs (■). MEI, kJ/kg BW^0.60^ d = 1.88 (±0.128)×ADG, g/d +782 (±72.848), R^2^ = 0.86, p<0.01, n = 36.

Linear regressions of MEI (kJ/kg BW^0.60^ d) against ADG (g/d) are presented in Figure 3. The regression equations (parameters ±SE) were MEI, kJ/kg BW^0.60^ d = 1.88 (±0.128) ×ADG, g/d+ 782 (±72.848), R^2^ = 0.86, p<0.001. The calculated ME_m_ in this study was 782 kJ/kg BW^0.60^ d from Figure 3. Specifically, combined with the equations for energy retained as protein and fat, if the ME supplied to growing pigs is about 782 kJ/kg BW^0.60^ d, protein retention of pigs would be positive while fat retention of pigs would be negative.

## DISCUSSION

### Growth performance and nutrient utilization

Increasing feed intake increased not only MEI, but also other nutrients such as protein. Thus, effects of feeding level on ATTD of energy and nutrients were determined. The ATTD of DM, GE, and CP were not affected by an increase in feeding level. This result is supported by the research of Peers et al [24] who assessed the digestibility of DM, GE, and total nitrogen in barley and some of the findings of Zhang et al [14] who estimated the effects of previous feeding level on nutrient utilization in pigs. However, our results were in contrast with the observations of Goerke et al [9] that the ATTD of DM, CP, organic matter, ash, and GE decreased in diets as the feeding level increases. The relationship between feeding level and digestibility of nutrients appears to depend on the characteristic of the feed [10]. Dietary fiber is one of the main components in the diet which affects digestibility because higher dietary fiber is inefficiently degradative and makes digesta rapidly pass through the gastrointestinal tract [24,25]. In the present study, the type of dietary fiber was the same for all treatments and the concentration of fiber was lower than in the diets used by Goerke et al [9]. Therefore, this may be explained that dietary feeding level had no difference in the ATTD of DM, GE, and CP in the present study.

Digestible energy and ME of diets were not affected by the feeding level, which were in agreement with results obtained in previous studies [3,24]. Similarly, Lovatto et al [26] investigated the effects of feed restriction and subsequent refeeding on energy utilization in growing pigs, and no differences were observed in energy digestibility during feed restriction. Different feeding levels had no effect on the value of ME:DE ratio, which agrees with previous observations in growing pigs [9]. The ME:DE ratio is related to the protein content of the diet and the amount of nitrogen in urine [27,28]. In the current study, nitrogen excreted in urine as a proportion of nitrogen intake had no association with dietary energy intake, which may explain the results of the ME:DE ratio [25].

The ADG in the present study was 773 g/d at the 100% feeding level, whereas ADG in previous studies ranged from 560 to 900 g/d [29–31]. The ADG improved as the feeding level increased. Similar results have been reported by Campbell and Dunkin [32] and Quiniou et al [33]. The findings of the current study suggested that the dietary energy content was higher than ME_m_ and anabolism exceeded catabolism [34].

### Energy and nitrogen balance

The growth of pigs is a dynamic process of quantitative inputs and outputs of carbohydrate, protein and fat, which can be measured by gas exchange calorimetry or carbon-nitrogen balance [35]. The HP is described as the energy lost and not retained in the ME [3,36]. In the present study, the HP of different feeding levels were mainly provided by OXCHO, which accounted for about 86% of the total HP, while the HP from OXP was only 11% of total HP. Similarly, Chwalibog et al [21] reported that the proportion of carbohydrate oxidation during feeding was about 90%, and protein oxidation accounted for only about 9%. Therefore, the HP and OXCHO increased as feeding level increases.

Energy retention is the fraction of ME retained in the body during feeding. The retention of protein and fat were 159 g/d and 133 g/d, respectively, when pigs were fed *ad libitum*. Fat deposition was slightly lower than Quiniou et al [33] and de Lange et al [3], which may be a result of the younger pigs used in the present study. However, if expressed as kJ/kg BW^0.60^ d, the value of RE_p_ was higher than fat at lower feeding levels, but lower at higher feeding levels. This may be explained by the fact that pigs give preference to use dietary energy to synthesize protein [34,37,38], and fat formation increases when energy intake is above the requirement for maintenance and maximal muscle formation [39]. Some equations that described the relationship between RE_p_ or RE_f_ and MEI have been established based on comparative slaughter [33,40]. However, considering that comparative slaughter is labor intensive and requires a large number of animals, indirect calorimetry was used for measurement of energy retention [41], though the latter generally estimated higher energy and protein retention than comparative slaughter [37]. From previous equations and the equations established in the current studies, daily RE_p_ increased linearly with MEI [33]. However, several studies have proposed that there was a linear-plateau relationship between energy intake and the rate of protein deposition [42], which were not determined in the current study. This could be partly explained that daily RE_p_ did not reach the maximum under the current *ad libitum* feeding level [43]. Meanwhile, the increase in RQ and RQ_np_ which was observed as MEI increased reflects the elevation in body fat gain [3,20].

### Serum biochemical parameters related to energy metabolism

The BHBA is one of the important intermediate metabolites of fatty acid oxidation in the liver [44]. Levels of BHBA increase in the liver, heart, muscle, brain, and other tissues with calorie restriction and fasting [45]. In the current experiment, the feeding level had no difference but there was a linear trend on the concentration of BHBA, which may be explained by the fact that the lowest feeding level fed to pigs was higher than ME_m_. Serum urea nitrogen concentration can be an indirect indicator of the AA utilization in the diet, as increases in SUN reflect reduced efficiency of nitrogen utilization and more synthesis of urea [46]. In the present research, SUN concentrations of pigs were also not different, which indicated that the efficiency of nitrogen utilization was not affected by the feeding level when the MEI of growing pigs was higher than ME_m_.

### Energy requirement for maintenance

Based on the linear regression analyses, the estimated ME_m_ in present study was 782 kJ/kg BW^0.60^ d. The ME_m_ was close to the value (774 kJ/kg BW^0.60^ d) reported by de Lange et al [3] who calculated ME_m_ from energy retention. Energy requirement for maintenance of other studies ranged from 720 to 1,120 kJ/kg BW^0.60^ d [1–3,47]. However, the ME_m_ was lower than the results of Wisesmuller et al [38], which may be explained by the fact that the content of dietary crude fiber in the current experiment was lower (2.75% vs 9.0%). As previously mentioned, when estimated ME_m_ was substituted into the equations of energy retention for fat and protein, protein retention of pigs would be positive while fat retention of pigs would be negative. Our results suggested that growing pigs tend to deposit protein at the expense of fat at estimated maintenance state. These results were consistent with the reports of Quiniou et al [33] who performed regression analysis between protein or lipid deposition and MEI above maintenance. Evidences suggested that energy utilization for maintenance is partly related to protein turnover because proteins are essential parts of organisms and perform a vast array of functions within cells [48,49]. However, fat is a main source to meet the energy requirements when the energy supply is restricted, which is a wise strategy for saving glucose and protein to important organs and tissues in the body [50]. But even so, it was a pity that serum biochemical parameters, which were associated with protein and fat metabolism, were little influenced by feeding level [45,46]. Therefore, further research is needed to reveal the metabolism for process of maintenance.

## CONCLUSION

In the present study, energy requirement for maintenance was estimated at 782 kJ/kg BW^0.60^ d based on linear regression analyses. This indicates that growing pigs will deposit protein and mobilize fat if MEI is close to estimated requirement for maintenance.

## Figures and Tables

**Table 1:** Ingredient and analyzed nutrient composition of the experimental diet (as-fed basis)

Item	Basal diet
Ingredient (%)	
Corn	71.00
Soybean meal, 43% CP	23.00
Wheat bran	2.70
Dicalcium phosphate	1.10
Limestone	0.95
L-lysine·HCl, 78%	0.40
Salt	0.35
Vitamins and minerals premix1)	0.50
Analyzed nutrient composition (%)	
Crude protein	15.88
Ether extract	2.66
Crude fiber	2.75
Calcium	0.68
Total phosphorus	0.55
Lysine	1.02
Methionine+cysteine	0.57
Tryptophan	0.17
Threonine	0.60
GE (MJ/kg)	16.02
ME (MJ/kg)2)	13.98

CP, crude protein; GE, gross energy; ME, metabolizable energy.

1) Vitamin-mineral premix supplied the following nutrients per kilogram of diet: vitamin A, 5,512 IU; vitamin D_3_, 2,200 IU; vitamin E, 30 IU; vitamin K_3_, 2.2 mg; vitamin B_12_, 27.6 μg; riboflavin, 4 mg; pantothenic acid, 14 mg; niacin, 30 mg; choline chloride, 400 mg; folic acid, 0.7 mg; thiamine, 1.5 mg; pyridoxine, 3 mg; biotin, 44 μg; Mn (MnO), 40 mg; Fe (FeSO_4_·H_2_O), 75 mg; Zn (ZnO), 75 mg; Cu (CuSO_4_·5H_2_O), 100 mg; I (KI), 0.3 mg; Se (Na_2_SeO_3_), 0.3 mg.

2) Metabolizable energy content of the diet was calculated using energy values for the ingredients obtained from NRC [51].

**Table 2:** Effects of feeding level on growth performance, apparent total tract digestibility of nutrients and energy and energy values of diets in growing pigs1)

Item	Feeding level (% of *ad libitum*2))						SEM	p-value3)	
	
	50	60	70	80	90	100		Linear	Quadratic
Initial BW (kg)	26.3	26.2	26.4	26.4	26.3	26.3	0.77	0.908	0.889
Final BW (kg)	33.5	37.3	40.8	46.0	46.8	49.9	0.89	0.002	<0.001
Growth performance									
Feed intake (kg/d)	0.66	0.82	0.98	1.17	1.31	1.51	0.01	<0.001	<0.001
Average daily gain (kg/d)	0.24	0.37	0.48	0.61	0.68	0.77	0.03	<0.001	<0.001
Gain:feed	0.36	0.46	0.49	0.53	0.52	0.50	0.03	0.025	0.107
ATTD (%)									
DM	88.5	89.1	89.6	88.6	88.6	89.6	0.5	0.720	0.973
GE	88.5	89.1	89.6	88.6	88.7	89.7	0.6	0.661	0.932
CP	86.5	86.8	87.8	86.7	86.1	88.2	1.0	0.714	0.802
Energy value									
DE (MJ/kg DM)	16.11	16.20	16.30	16.13	16.14	16.31	0.11	0.661	0.932
ME (MJ/kg DM)	15.76	15.85	15.88	15.84	15.81	16.04	0.13	0.511	0.694
ME:DE ratio	97.87	97.86	97.47	98.23	97.96	98.32	0.28	0.413	0.368
NE4) (MJ/kg DM)	11.87	11.73	11.78	11.44	11.09	11.32	0.51	0.345	0.928

ATTD, apparent total tract digestibility; SEM, standard error of the mean; BW, body weight; DM, dry matter; GE, gross energy; CP, crude protein; DE, digestible energy; ME, metabolizable energy; NE, net energy.

1) Data are means of 6 replicates per treatment.

2) The estimated *ad libitum* ME intake was 2,400 kJ/kg BW^0.60^ d.

3) Linear and quadratic contrasts for feeding level.

4) Net energy requirement for maintenance was obtained from Noblet et al [15].

**Table 3:** Effects of feeding level on energy and nitrogen balance and energy retention in growing pigs1)

Item	Feeding level (% of *ad libitum*2))						SEM	p-value3)	
	
	50	60	70	80	90	100		Linear	Quadratic
Energy balance (kJ/kg BW^0.60^ d)									
ME intake	1,207	1,440	1,671	1,906	2,116	2,368	12	<0.001	0.909
Total heat production	953	1,006	1,046	1,113	1,214	1,266	42	0.001	0.545
Oxidation of protein	106	112	119	145	139	172	23	0.206	0.705
Oxidation of carbohydrate	824	876	895	936	1,042	1,057	49	0.023	0.713
Nitrogen balance (g/d)									
Intake	18.2	21.7	25.3	29.0	33.1	36.6	0.4	<0.001	0.547
Fecal excretion	2.4	2.8	3.0	3.4	4.6	3.9	0.4	0.010	0.585
Urinary excretion	7.3	7.7	8.6	10.0	10.3	13.2	1.6	0.109	0.505
Retention	8.5	11.2	13.7	15.6	18.2	19.6	1.6	0.002	0.673
Energy retention (kJ/kg BW^0.60^ d)									
RE_p_	152	205	249	269	318	332	29	0.006	0.502
RE_f_	116	254	398	534	613	701	51	<0.001	0.290
Total	268	459	648	803	932	1,033	44	<0.001	0.103
Respiratory quotient	1.02	1.03	1.06	1.06	1.08	1.08	0.01	0.006	0.135
Non-protein respiratory quotient	1.02	1.04	1.08	1.10	1.11	1.12	0.01	0.001	0.099

SEM, standard error of the mean; BW, body weight; ME, metabolizable energy; RE_p_, energy retained as protein; RE_f_, energy retained as fat.

1) Data are means of 6 replicates per treatment.

2) The estimated *ad libitum* ME intake was 2,400 kJ/kg BW^0.60^ d.

3) Linear and quadratic contrasts for feeding level.

**Table 4:** Effects of feeding level on the concentration of serum biochemical parameters in growing pigs1)

Item	Feeding level (% of *ad libitum*2))						SEM	p-value3)	
	
	50	60	70	80	90	100		Linear	Quadratic
Glucose (mmol/L)	4.63	5.52	4.96	5.12	5.10	5.07	0.31	0.700	0.767
Urea nitrogen (mmol/L)	1.53	2.38	1.71	1.77	1.87	2.25	0.35	0.543	0.771
Creatinine (mmol/L)	56.42	73.21	65.39	58.73	60.75	59.18	7.83	0.917	0.340
Triglyceride (mmol/L)	0.23	0.22	0.21	0.22	0.22	0.29	0.04	0.301	0.284
Free fatty acids (mmol/L)	0.53	0.50	0.48	0.41	0.49	0.51	0.04	0.175	0.761
β-hydroxybutyric acid (mmol/L)	0.13	0.11	0.13	0.12	0.12	0.13	0.01	0.080	0.889
Leptin (μg/L)	6.63	6.82	6.64	6.75	8.07	6.98	0.56	0.238	0.129

SEM, standard error of the mean.

1) Data are means of 6 replicates per treatment.

2) The estimated *ad libitum* metabolizable energy intake was 2,400 kJ/kg BW^0.60^ d.

3) Linear and quadratic contrasts for feeding level.
